# Rare disease day and Ophthalmology

**DOI:** 10.5935/0004-2749.202100106

**Published:** 2021

**Authors:** Mariana Matioli da Palma, Cecilia Francini Cabral de Vasconcellos, Juliana Maria Ferraz Sallum

**Affiliations:** 1 Department of Ophthalmology, Escola Paulista de Medicina, Universidade Federal de São Paulo, São Paulo, SP, Brazil; 2 Instituto de Genética Ocular, São Paulo, SP, Brazil

Dear Editor,

Globally, across the six continents, February 28^th^ 2021 was celebrated as Rare Disease Day. The goal of this day is to generate awareness in the public and health professionals on the effects of these rare diseases on patients and their families. Many patients are struggling, searching for diagnoses and appropriate treatments^(1)^.

By definition, a rare disease is a health condition that affects up to 65 individuals per 100,000, or 1.3 per 2,000 ^(2)^. It is estimated that out of a global population of approximately 7.8 billion people, roughly 300 million have a rare disease^(1)^. Most are severe, chronic, and life-threatening. There are approximately 7,000 rare disea ses in the world^(2)^, and some are even classified as ultra-rare (less than or equal to 1:50,000 inhabitants according to *Conselho Nacional de Saúde*. Resolucion 563)^(3)^.

Rare diseases include rare forms of cancer, infectious diseases, autoimmune diseases, and genetic disorders. Approximately 80% of rare diseases have a genetic origin^(2)^; most start in childhood^(2)^. Importantly, many rare diseases affect ocular tissue. Retinoblastoma is a rare form of retinal cancer in young children but has a high survival rate^(4)^. Congenital cataracts are a treatable cause of visual impairment and blindness during infancy. The estimated prevalence is 4.24 per 10,000 individuals making it a rare disease. Zika virus may lead to a rare congenital Zika virus syndrome with typical chorioretinal atrophy in the macula area. Mucopolysaccharidoses are rare metabolic disorders characterized by corneal deposition, leading to visual impairment. A differential diagnosis for corneal clouding is the rare congenital glaucoma. Inherited retinal dystrophies (IRD) are a group of ocular diseases marked by high clinical and genetic heterogeneity affecting approximately 1 in every 2,000-3,000 individuals^(5)^. IRDs are rare diseases that may be isolated or associated with syndromic conditions^(5)^. In Brazil, it is estimated that out the 210 million inhabitants, at least 70,000 have IRDs.

Genetic testing contributes to precision medicine, accurate diagnoses, and improved genetic counseling. As genetic treatments are approved and developed, genetic testing has gained considerable importance. The first gene therapy approved by the Food and Drug Administration in 2017 was Luxturna for IRD patients with biallelic *RPE65* mutations^(6)^. This medication was also approved in Brazil by *Agência Nacional de Vigilância Sanitária*. The *RPE65* gene causes Leber congenital amaurosis and retinitis pigmentosa, and positively, the Luxturna treatment changed the natural history of the disease improving vision as shown in the multi-luminance mobility test^(6)^. Genetic testing is essential if an individual is to be considered a candidate for gene therapy surgery. Furthermore, IRD based clinical trials are ongoing and are focused on gene replacement, RNA-based therapies, or editing receptor’s genetic material^(7)^. All of this together reinforces the importance of improving knowledge of rare IRDs.

IRDs can also be associated with syndromic conditions. Senior-Loken is one example of an ultra-rare ocular syndrome where the retina is affected first by severe early-onset Leber congenital amaurosis or more typical retinitis pigmentosa. Renal abnormality is characterized by nephronophthisis which may progress to end-stage renal disease during early life in these patients. The ophthalmologist must be aware of this possibility indicating work-up for renal involvement and therefore preventing renal failure. It is a challenge to assist patients with inherited eye diseases, a correct early diagnosis combined with appropriate molecular testing can provide optimal interdisciplinary follow-up outcomes.

This strategy is facilitated by a multidisciplinary team approach involving, nurses, support workers, careers, social workers, nutritionists, pediatricians, ophthalmologists, geneticists, psychologists, and patient organizations^(1)^. Many aspects of daily life are impacted by rare eye diseases, including disability acceptance, academic life, work-life, financial conditions, daily living activities, and mobility. In addition, some IRDs exert different impacts on other aspects of daily life, e.g., night vision difficulties for patients with nyctalopia, or simple visual difficulties in dim environments. Other IRDs generate difficulties for patients with photophobia in sunny environments, while some IRDs related to central vision loss impose difficulties for interpersonal relationship e.g. maintaining eye contact during conversations. Thus, difficulties during social interactions could lead to emotional issues.

Families, health professionals, and patient associations must work together to support patients with rare and ultra-rare diseases. Each year on the last day of February^(1)^, the rare disease community comes together to remind everyone of ongoing progress, and importantly, how much works still needs to be done.
